# Assessment of the general public's knowledge about rheumatic diseases: evidence from a Portuguese population-based survey

**DOI:** 10.1186/1471-2474-11-211

**Published:** 2010-09-16

**Authors:** Milton Severo, Rita Gaio, Raquel Lucas, Henrique Barros

**Affiliations:** 1 Department of Hygiene and Epidemiology, University of Porto Medical School, Porto, Portugal; 2 Institute of Public Health of the University of Porto, Porto, Portugal; 3 Department of Mathematics, University of Porto Science School; Center of Mathematics of the University of Porto

## Abstract

**Background:**

To identify incorrect beliefs and common knowledge about rheumatic diseases in the general population.

**Methods:**

Participants were selected during the follow-up of a representative cohort of adult population of Porto, Portugal; 1626 participants completed a questionnaire that included general knowledge items about rheumatic diseases.

Discrete and continuous latent variable models were used to identify knowledge flaws and the target groups. Odds ratios (OR) estimated by multinomial logistic regression, and 95% confidence intervals (95%CI) were computed to evaluate magnitude of associations.

**Results:**

A continuous latent variable model identified two dimensions: one related to general beliefs (latent 1) and another concerning characteristics, treatment and impact of rheumatic diseases (latent 2). A 3-class latent variable model refined these results: the first class presented the lowest probabilities of correct answer for items associated with the first latent (mean of 39%), and the second class presented the lowest probabilities of correct answer for items with the second latent (mean of 62%). The third class showed the highest probability of a correct answer for almost all the items (mean of 79%). The age and sex standardized prevalence of the classes was 25.7%, 30.8% and 43.5%.

Taking class 2 as reference, class 1 was positively associated with the presence of rheumatic diseases (OR = 2.79; CI95% = (2.10-3.70)), with females (OR = 1.28 CI95% = (0.99-1.67)) and older individuals (OR = 1.04; CI95% = (1.03-1.05)), and was negatively associated with education (OR = 0.84; CI95% = (0.81-0.86)); class 3 was positively associated with education (OR = 1.03; CI95% = (1.00-1.05)) and the presence of rheumatic diseases (OR = 1.29; CI95% = (0.97-1.70)).

**Conclusions:**

There are several knowledge flaws about rheumatic diseases in the general public. One out of four participants considered false general beliefs as true and approximately 30% did not have detailed knowledge on rheumatic disease. Higher education and the presence of disease contributed positively to the overall knowledge. These results suggest some degree of effectiveness of patient education, either conducted by health professionals or self-driven.

## Background

Musculoskeletal diseases are among the most prevalent chronic conditions and constitute a major public health challenge for our aging societies [1]. Providing the general population and patients with good quality information is an important strategy in the management of chronic diseases. Knowledge leads to changes in attitudes and behaviours, and directly influences health status [2], and adequate information can promote self-management skills necessary for coping with the disease increasing adherence to therapy [3].

Patient participation in health care has been increasingly advocated: patients should be well informed about diagnosis and prognosis, and involved as fully as possible in disease management, namely in therapeutic decisions. A partnership should be formed between patients and health professionals, especially regarding chronic or life threatening diseases [4]. Involvement in medical decisions has been positively associated with patient satisfaction with health care [2,5] and improved health outcomes [6]. In rheumatoid arthritis cases, patient education has a positive effect in adherence to treatment, functional disability, global assessment, psychological well-being and depression [7,8].

Several studies showed that requirements for information are associated with patients age and education [9] but the overall level of information about rheumatic diseases is low among patients living with these conditions [9-11]. Although research targeting the general population is scarce, a survey of the Dutch population showed similar results [12] and raised the need for the identification of dimensions involved in knowledge about rheumatic diseases and the quantification of common knowledge in each specific demographic, social or pathology group. If nothing else, such quantification would benefit an education program targeted to musculoskeletal health.

By using a previously developed questionnaire designed to evaluate the overall knowledge level about rheumatic diseases in the general population [12], we aim to identify the incorrect beliefs and common knowledge about rheumatic diseases in a sample of the general population and to identify target groups for health education.

## Methods

Participants were selected during the follow-up, conducted in 2005-2008, of a representative cohort of the non-institutionalized adult population of Porto, Portugal - the EpiPorto cohort. Recruitment at baseline was done using random digit dialling [13], selecting a single person over 17 years old in each of the identified households. Trained interviewers collected information, using a standard protocol that comprised multiple exams and questionnaires. Besides questions on social, demographic, clinical and behavioural characteristics participants completed an interviewer-administered questionnaire, comprising 17 statements about rheumatic diseases to be considered true or false. This was the Portuguese version of a Dutch questionnaire designed to evaluate knowledge regarding rheumatic diseases [12].

Cultural validation of the Portuguese version of the scale followed the usual methodology. The first stage consisted of a forward translation completed by 2 independent professional translators, yielding 2 initial Portuguese versions. Translators then synthesized the 2 versions to create a consensus version. Afterwards, 2 different independent translators completed a backward translation. Finally, an expert committee reviewed and compared the final Portuguese translation and the back translations to obtain a final version of the scale.

History of chronic rheumatic disease was self-reported, each individual indicating whether he/she had ever been diagnosed, by a doctor, with rheumatoid arthritis, ankylosing spondylitis, psoriatic arthritis, hand, hip or knee osteoarthritis, osteoporosis or lupus.

At baseline, 2485 participants were recruited, of whom 82 (3.3%) died before follow-up, 199 (8.0%) refused to be re-evaluated and 578 (23.3%) were unreachable by telephone or post. Therefore data from 1626 (65.4%) individuals were available for the present study. They presented a mean age of 58 (±15) years and 9 (±5) years of education; 1014 (62.4%) were women and 528 (32.6%) reported having been diagnosed with at least one rheumatic disease (table 1).

**Table 1:** Sample characteristics: socio-demographics information and history of rheumatic disease


	**N (%)**

**Gender**	

Women	1014 (62.4)
Men	612 (37.6)

**Self-report Rheumatic Diseases**	n (%)

Any	528 (32.6)
Rheumatoid arthritis	48 (3.0)
Ankylosing spondylitis	24 (1.5)
Hand osteoarthritis	211 (13.0)
Hip osteoarthritis	117 (7.2)
Knee osteoarthritis	247 (15.2)
Osteoporosis	265 (16.3)
Other	5 (0.3)

**Age (years)**	mean ± SD

	58 (14)

**Education level (years)**	9 (5)

The local ethics committee (Hospital São João) approved the study protocol. All participants gave informed written consent to participate in the study, which was carried out in accordance with the Helsinki Declaration.

### Statistical Analysis

Latent variable models were used to identify the incorrect beliefs and common knowledge about rheumatic diseases in the general population and to identify specific target groups.

In the present study, and given the binary structure of the data, two models were used: latent trait models (LTM) and latent class models (LCM).

LTM was used to identify dimensions in knowledge about rheumatic diseases in the general population, thus identifying what we considered to be incorrect beliefs and common knowledge. LTM assume that the performance of an individual while answering the items is explained by one or more (continuous) variables, commonly called "latent variables". LTM is simply a Binary Data Factor Analysis that considers one or more factors.

Interpretation of the model is usually done considering the standardized factor loadings. Each of these expresses the correlation coefficient between the latent variable and an underlying continuous variable obtained from each item [14]. An association is classified as weak if the corresponding standardized loading is less than 0.30, moderate if it is between 0.30 and 0.70, and strong if it is higher than 0.70. Varimax rotation was applied to simplify the standardized factor loadings matrix.

LCA was used to uncover heterogeneous groups of individuals, thereby identifying the target groups. Latent class models (LCM) consider that the performance of an individual on the items is explained by *K *classes, commonly called "latent classes". Interpretation of the model is usually done by looking at the probabilities of positive response on each item conditional on class membership.

The global *goodness of fit *of the considered latent models was assessed through the likelihood ratio test, via parametric boostrapping - 100 samples - given the sample size and the number of estimated parameters [15]. Marginal *goodness of fit *was also evaluated through residuals inspection. The number of latent variables or classes in the considered LTM or LCM was the smallest providing the best *goodness of fit *to the given data. Correspondence analysis and principal component analysis from the item underlying continuous variables were also applied to confirm that number. Once the latent variables in the LTM were extracted, standardized Cronbach's alpha was estimated from the polychoric correlations between two binary variables [16], inter-item (tetrachoric) correlations mean and item-total biserial correlation coefficient [17] were used to evaluate the internal consistency of the group of items defining each variable.

Odds ratios (OR), estimated by multinomial logistic regression, and their respective 95% confidence intervals (95%CI) were used to measure the magnitude of associations between latent classes and the covariates sex, age, education and self-reported rheumatic diseases.

The distribution of the sample by latent classes was standardized by sex and 10-year age bands according to the 2001 census counts for the city of Porto. Significance level was fixed at 0.05.

Statistical analyses were performed using the software R 2.8.1 [18], and specifically, the ltm and lca command from, respectively, the ltm [19] and e1071 [20] packages.

## Results

Among the 1626 participants, 1449 (95.9%) answered all the statements. The proportion of individuals that correctly answered each item ranged from 28% to 93%, corresponding to items 13 and 5, respectively, and the mean of correctly answered items was 10.5 (± 2.3) (table 2). The item-total biserial correlation coefficient computed for each item ranged from 0.31 to 0.60, corresponding to items 5 and 14, respectively. Cronbach's alpha was 0.628 and the inter-item correlation mean was 0.09.

**Table 2:** Proportion of correct answers and respective 95% confidence interval (95%CI) for each statement, standardized loadings for the 2-factor latent trait model (LTM) and probability of correct answer in the 2 and 3-classes latent class model (LCM)

			LTM		2-classes LCM		3-classes LCM		
**Statement (correct option)**	**Proportion of correct answers**	**One Factor**	**Two factor Model**		**Class 1 (52%)**	**Class 2 (48%)**	**Class 1 (31.1%)**	**Class 2 (28.1%)**	**Class 3 (40.9%)**

	% (95%CI)	**Std.z1**	**Std.z1**	**Std.z2**	%	%	%	%	%

1. A rheumatic disease is especially characterised by pain and stiffness in muscles and joints (t)	82 (80-84)	0.137	0.109	**0.315**	82	83	**84**	0.74	**88**

2. Rheumatic diseases are only seen in older women (f)	87 (85-89)	**-0.871**	**0.872**	0.000	99	74	68	**92**	**100**

3. In general, rheumatic patients should rest as much as possible and move as little as possible (f)	82 (80-83)	**-0.735**	**0.703**	0.060	95	68	63	**85**	**95**

4. Almost all rheumatic patients will finally end up in a wheelchair (f)	71 (68-73)	**-0.866**	**0.753**	-0.303	97	42	29	**86**	**94**

5. Medications for osteoarthritis cannot cure the disease, but can relieve pain and stiffness (t)	93 (92-95)	**0.529**	-0.153	**0.548**	91	96	**97**	87	**96**

6. Glandular fever is a kind of rheumatic disease (f)*	27 (25-29)	-**0.408**							

7. Rheumatoid arthritis is a rheumatic disease in which the joints are affected with inflammations (t)	89 (87-90)	0.027	0.265	**0.581**	90	88	**90**	79	**96**

8. Affected joints of rheumatic patients can be replaced with artificial joints (t)	54 (52-57)	0.285	-0.089	**0.333**	48	61	**65**	41	**56**

9. Osteoarthritis (wear and tear) is the most common kind of rheumatic disease (t)	88 (87-90)	**0.525**	-0.068	**0.619**	85	92	**95**	72	**97**

10. Multiple sclerosis (MS) is a rheumatic disease (f)*	37 (34-39)	**-0.655**							

11. People can die from the consequences of rheumatic disease (t)	47 (44-49)	0.200	-0.003	0.170	45	47	**49**	38	**51**

12. No kinds of rheumatic diseases can be cured (f)	37 (35-39)	**-0.387**	**0.332**	0.062	45	29	27	**34**	**49**

13. Rheumatoid arthritis is caused by poor diet, and cold and damp weather (f)	28 (25-30)	-**0.680**	**0.529**	-0.099	41	13	08	**34**	**39**

14. Ankylosing spondylitis is a kind of rheumatic disease (t)*	47 (45-50)	0.131							

15. There are more than 100 different kinds of rheumatic diseases (t)	66 (63-68)	**0.391**	-0.054	**0.520**	60	72	**78**	42	**76**

16. About one out of every twenty Portuguese people is being treated for a rheumatic disease (t)	84 (82-86)	**0.613**	-0168	**0.765**	77	92	**97**	59	**95**

17. Fibromyalgia is a rheumatic disease (t)*	38 (36-40)	0.137							

*eliminated from the model

### Latent Trait Model

As no prior information on the number of latent variables to be held was available, a one-factor LTM was fit to the 17 items. Seven items showed a moderate-to-strong negative association with the latent variable while four presented a moderate positive association (table 2). A global test of *goodness-of-fit *(G^2 ^= 1901, p < 0.01) enhanced by the inspection of 2 by 2 marginal residuals showed a poor fit of this model.

A two-factor LTM with a varimax rotation presented moderate-to-strong associations between seven items and the first latent variable, and between nine items and the second latent variable. One item showed a weak association with the extracted factors. This model also presented a poor fit (G^2 ^= 5945, p < 0.01) and the inspection of marginal residuals revealed large pairwise residuals for four items (6, 10, 14 and 17) whose statement followed the structure "... is a kind of rheumatic disease." The *goodness-of-fit *was improved after elimination of those items (G^2 ^= 1901, p = 0.18) (table 2). This final model associates items 2, 3, 4, 12 and 13 with the first latent variable (LT1), and items 1, 5, 7, 8, 9, 15 and 16 with the second one (LT2), providing a standardized alpha of 0.700 and 0.630 and inter-item correlation of 0.32 and 0.20, respectively.

### Latent Class Model

A latent class model with three classes was fit (G^2 ^= 2027.525, p > 0.99) to the 13 items considered in the above 2-factor LTM.

The first class presented the lowest probabilities of correct answer for items associated with the first latent (mean of 39%), and the second class presented the lowest probabilities of correct answer for items with the second latent (mean of 62%). The third class showed the highest probability of a correct answer for almost all the items (mean of 79%).

Four hundred and ninety individuals (31.0%) were classified in the first latent class, 443 (28.1%) in the second class and 645 (40.9%) in the third class.

The multinomial logistic regression showed that class, 1 when compared with class 2, was positively associated with the presence of rheumatic disease (OR = 2.79; CI95% = (2.10-3.70)) with female gender (OR = 1.28 CI95% = (0.99-1.67)) and older age (OR = 1.04; CI95% = (1.03-1.05)) and negatively associated with education (OR = 0.84; CI95% = (0.81-0.86)). Class 3, compared with class 2, was positively associated with education (OR = 1.03; CI95% = (1.00-1.05)) and with history of rheumatic disease (OR = 1.29; CI95% = (0.97-1.70)) (table 3).

**Table 3:** Multinomial logistic regression model for latent classes by gender, age, education level, and self-report rheumatic diseases

	Class 3	Class 1	Class 3	Class 1
	**Crude OR (95CI%)**	**Crude OR (95CI%)**	OR** (95CI%)	OR** (95CI%)

Sex				

Women	1.41 (1.10-1.80)	1.28 (0.99-1.67)	1.32 (1.02-1.72)	0.92 (0.68-1.25)
Men	1	1	1	1

Age (years)	1.00 (0.99-1.00)	1.04 (1.03-1.05)	1.00 (0.99-1.01)	1.01 (1.00-1.02)

Education level (years)	1.03 (1.00-1.05)	0.84 (0.81-0.86)	1.03 (1.00-1.06)	0.86 (0.83-0.89)

Self-reported Rheumatic Pathologies				

None	1	1	1	1
At least one	1.29 (0.97-1.70)	2.79 (2.10-3.70)	1.34 (0.98-1.85)	1.77 (1.27-2.47)

*latent class 2 as reference; **adjusted for all variables

After adjustment for all variables, age and gender effect were attenuated, while education and the history of rheumatic disease as the major determinants.

The age and sex standardized prevalence of latent classes were 25.7%, 30.8% and 43.5% in classes 1, 2 and 3, respectively.

## Discussion

This survey revealed limited knowledge regarding rheumatic diseases at the general population level: there were difficulties regarding the identification of whether diseases where rheumatic (ankylosing spondylitis and fibromyalgia) or not (glandular fever and multiple sclerosis), and more than fifty percent believed that people with rheumatic diseases cannot be cured and cannot die from those illnesses (table 2). The latter finding is similar to that reported in other studies: in Canada [21] a study on women aged 65-90 years showed that only 36% agreed that health problems caused by osteoporosis can be life-threatening and another study carried out in US adults [22] found that only 63% correctly answer "false" to the statement "No medications can treat osteoporosis".

Considering these results, it is important to separate these knowledge domains in order to identify possible knowledge flaws and understand educational needs. Summarizing rheumatic diseases knowledge as a single domain is very limited. A recent review [21,23] identified significant limitations and constraints in measuring osteoporosis knowledge as a single domain, as it should include multi-dimensional aspects like causes or risk factors, prevention, consequences and treatment. A similar situation holds for the present questionnaire when one tries to summarize rheumatic diseases knowledge after a single value obtained from the 17 statements. The *goodness-of-fit *test suggest that the 2-factor LTM (13 items) is the best solution. The first latent was associated with the following statements, which probably represent wrong general beliefs: rheumatic diseases are more frequent in older women, rheumatoid arthritis is caused by poor diet, cold and damp weather, rheumatic patients should rest and move as little as possible and rheumatic diseases cannot be cured, and all rheumatic patients end up in wheelchairs. Items about aetiology, treatment and impact of the rheumatic diseases were related with second latent - which reveals specific knowledge.

The 3-class LCM showed that 25.7% agreed with the false general beliefs but did have specific knowledge (Class 1), 30.8% did not agree with the general beliefs and did not have specific knowledge (Class 2) and 43.5% did not agree with general beliefs and had specific knowledge (Class 3). Overall this suggests that almost 60% of the individuals had some flaws in their overall knowledge about rheumatic diseases.

**Figure 1:**
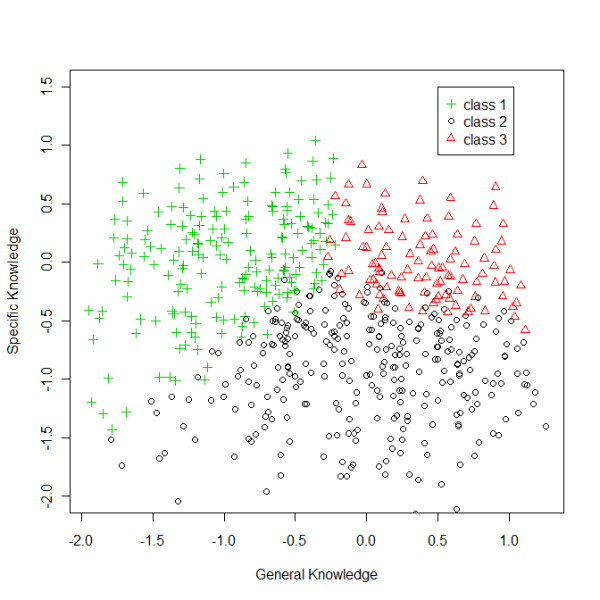
**The latent classes of the 3-latent class model allocated in the 2 dimensions (general and specific knowledge) of 2-factor latent trait model (13 items)**.

Considering this relationship between the 3-class LCM and 2-factor LTM (Figure 1), we expected that a history of at least one rheumatic disease was the major determinant of the first class, since the prevalence of rheumatic diseases is higher in women and in older individuals, and we also expected that this association would be extended to sex and age; for the second class, the expected major determinant would be education; and for the third class the presence of both characteristics: rheumatic disease history and higher educational level. The multinomial logistic regression confirmed these expectations: class 1 when compared with class 2 was positively associated with the history of rheumatic disease, female sex and old age and negatively associated with education; and class 3 when compared with class 2 was positively associated with education and the history of rheumatic disease. As expected, after adjustment for the presence of disease and education, the effect of age and sex was attenuated. Therefore the major determinants of knowledge were education and the presence of rheumatic diseases. Although we did not measure the effectiveness of patient education, these results suggest some degree of effectiveness of such, either conducted by health professionals or self-driven.

The effectiveness of futures educational programs about rheumatic diseases directed to general population/patient population might be improved by targeting the eldest and low educated fraction of the population to counteract wrong general beliefs. As reported in other studies education is not one programme, but a strategy that is tailored to each population, the programme should remodel the interpretative structures of individuals because providing educational information, by itself, has no beneficial impact [24].

There are a number of limitations to this study. First, this sample of the study was significantly older and had higher frequency of women when compared with census counts for the city of Porto, which could lead to a selection bias. However we have tried to minimize this by estimating the age and sex standardized prevalence of latent classes. Additionally, we used only a pool of 13 items to indentify incorrect beliefs and common knowledge and targets groups in the overall knowledge about rheumatic diseases. This is somehow limited, as the moderated alpha shows, considering that we are trying to measure a multi-dimensional concept, with a multitude of possible items.

## Conclusions

The use of latent models applied to this specific scale, we were able to provide evidence for identification of different knowledge domains regarding rheumatic diseases in the general population. Additionally, this method was instrumental to identify relevant target groups for educational programmes.

This study showed that there are several knowledge flaws about rheumatic diseases in the general population. One out of four considered the false general beliefs as true and approximately 30% did not have detailed knowledge on rheumatic disease. Higher education and the presence of disease contributed positively to the overall knowledge. However there is a major flaw in identifying what is and what is not a rheumatic disease in the general population.

## Competing interests

The authors declare that they have no competing interests.

## Authors' contributions

MS participated in the study design, performed the statistical analysis and helped to draft the manuscript. RG performed the statistical analysis and helped to draft the manuscript. RL and HB participated in the study design and helped to draft the manuscript.

All authors read and approved the final manuscript

## Pre-publication history

The pre-publication history for this paper can be accessed here:

http://www.biomedcentral.com/1471-2474/11/211/prepub
